# Latitudinal Range Influences the Seasonal Variation in the Foraging Behavior of Marine Top Predators

**DOI:** 10.1371/journal.pone.0023166

**Published:** 2011-08-10

**Authors:** Stella Villegas-Amtmann, Samantha E. Simmons, Carey E. Kuhn, Luis A. Huckstadt, Daniel P. Costa

**Affiliations:** 1 Department of Ecology and Evolutionary Biology, University of California Santa Cruz, Santa Cruz, California, United States of America; 2 Department of Ocean Sciences, University of California Santa Cruz, Santa Cruz, California, United States of America; Institut Pluridisciplinaire Hubert Curien, France

## Abstract

Non-migratory resident species should be capable of modifying their foraging behavior to accommodate changes in prey abundance and availability associated with a changing environment. Populations that are better adapted to change will have higher foraging success and greater potential for survival in the face of climate change. We studied two species of resident central place foragers from temperate and equatorial regions with differing population trends and prey availability associated to season, the California sea lion (*Zalophus californianus*) (CSL) whose population is increasing and the endangered Galapagos sea lion (*Zalophus wollebaeki*) (GSL) whose population is declining. To determine their response to environmental change, we studied and compared their diving behavior using time-depth recorders and satellite location tags and their diet by measuring C and N isotope ratios during a warm and a cold season. Based on latitudinal differences in oceanographic productivity, we hypothesized that the seasonal variation in foraging behavior would differ for these two species. CSL exhibited greater seasonal variability in their foraging behavior as seen in changes to their diving behavior, foraging areas and diet between seasons. Conversely, GSL did not change their diving behavior between seasons, presenting three foraging strategies (shallow, deep and bottom divers) during both. GSL exhibited greater dive and foraging effort than CSL. We suggest that during the warm and less productive season a greater range of foraging behaviors in CSL was associated with greater competition for prey, which relaxed during the cold season when resource availability was greater. GSL foraging specialization suggests that resources are limited throughout the year due to lower primary production and lower seasonal variation in productivity compared to CSL. These latitudinal differences influence their foraging success, pup survival and population growth reflected in contrasting population trends in which CSL are more successful and potentially more resilient to climate change.

## Introduction

As Darwin [1] stated: “It is not the strongest of the species that survive, nor the most intelligent, but the one most responsive to change”, thus species that are more capable of adapting to changing environmental conditions will have a greater capability of responding to long term changes in climate. The physical environment changes at different scales; over centuries, decades, years and seasonally within a year. Seasonal changes in temperature and light level, affect primary production and the abundance, distribution and behavior of higher trophic levels. Many taxa have evolved behavioral and physiological responses to avoid unsuitable seasonal change by migrating (whales and birds) [2], [3] or by means of hibernation and aestivation (bears, badgers and frogs) [4]–[6]. Life history patterns of other animals such as penguins, albatrosses, seals and sea lions; described as central place foragers [7], [8], respond to seasonal change by shifting their diet and/or foraging behavior.

The magnitude of seasonal change differs with latitude; it is more pronounced at higher latitudes than in equatorial regions, where there are typically only two seasons: a wet and a dry one. In equatorial regions environmental temperature remains high year round, there is a constant 12 hr period of daylight throughout the year and oceanic primary productivity is lower [9] (http://www.marine.rutgers.edu). At higher latitudes, colder marine systems are typically more productive and thus have a greater abundance of prey [9]. These latitudinal differences influence animal populations in many ways, e.g. foraging efficiency of Australian forest mammals is lower in tropical than in temperate ecosystems [10], tropical island terns reduce clutch investment at lower latitudes [11] and pinnipeds (seals, fur seals and sea lions) living in equatorial and temperate environments are more at risk of extinction than species living at higher latitudes [12]. These studies conclude that latitudinal differences in population size result from lower food availability and lower oceanic primary productivity, and prey depletion associated with resident-central place foraging behavior along with the reduced seasonality at lower latitudes.

Of the 15 Otariidae (fur seals and sea lions) species, population size declines with decreasing latitude with only two species in equatorial regions, a fur seal and a sea lion. Here we study the foraging behavior of two species of year round resident central place foragers, the temperate California sea lion (*Zalophus californianus*) (CSL), whose population is increasing (237–244,000 indiv.) [13], [14]; and the equatorial Galapagos sea lion (*Zalophus wollebaeki*) (GSL), whose population is endangered and declining (16–18,000 indiv.) [15], [16]. Given that CSL forage at higher latitudes where productivity and seasonality are greater, we hypothesized that CSL would exhibit a greater variability in their foraging behavior that is associated with the greater range of oceanic productivity they encounter.

Foraging behavior has been studied in an array of air breathing diving vertebrates such as penguins, seals [17] and all extant sea lion species [18]–[25]. However, all of these studies were conducted during one season, mostly summer. Seasonal dive behavior has only been studied in New Zealand (*Arctocephalus forsteri*), [26] and Subantarctic (*Arctocephalus tropicalis*) fur seals [27], [28], and in Steller (*Eumetopias jubatus*) and Australian (*Neophoca cinerea*) sea lions [18], [25], all of these showing greater effort during winter than summer. Villegas-Amtmann et al. [22] and Villegas-Amtmann & Costa [29] described 3 diving strategies in GSL that persisted over both a warm and cold season. The 3 groups from the cold season were classified in the same manner as the warm season with a minor modification in group 2 as follows: (1) shallow divers, sea lions that exhibited the shortest dive duration and shallowest dive depth, (2) deep bottom divers, individuals with the deepest dive depths, longest time at the bottom of a dive and mostly benthic (only “deep divers” during warm season as they were diving mesopelagically) and (3) bottom divers, sea lions with the highest percentage of benthic dives [29].

To determine how temperate and equatorial diving vertebrates with contrasting population status, respond to environmental change we compared CSL (Granito Island, Gulf of California, Mexico) and GSL (Caamaño Islet, Galapagos, Ecuador) diving behavior during two contrasting seasons, a warm and a cold one. Sea surface temperature (SST) around Granito Island ranges from 15–31°C and chlorophyll-a ranges from 0.3–6.0 mg/m^3^ , while around Caamaño Islet SST ranges from 19–28°C and chlorophyll-a from 0.3–0.8 mg/m^3^ during different seasons each year (http://coastwatch.pfeg.noaa.gov/). Additionally, we studied the differences in their diet by measuring carbon (^13^C/^12^C) and nitrogen (^15^N/^14^N) isotope ratios.

## Methods

### Ethics Statement

This research was approved by the CARC (Chancellor's Animal Research Committee) at University of California, Santa Cruz. Permission to import and collect samples was granted through National Marine Fisheries Service Permit No. 960-1528-00/PRT-017891 and No. 87-1593-06, Parque Nacional Galapagos authorization No. 084/06 PNG and SEMARNAT authorization No. SGPA/DGVS/06537 and 08736 and No. 09/FS-1837/01/07.

### Field site and tagging procedures

**California sea lions (CSL).** Research was carried out during a warm season- Jul-Aug 2005 and a cold one- Feb-Mar 2007 (seasons were defined as the contrasting ambient and water temperatures that occur each year) at Granito Island (29.55°N, 113.54°W) in the Gulf of California, Mexico. During the warm season (pupping season) we captured 10 lactating female CSL, which were suckling small pups, 1 to 2 months after peak pupping season. During the cold season (non-pupping season) we captured 11 lactating female CSL, with older pups (8 to 9 months after peak pupping season). Sea lions were captured with hoop nets and anesthetized with isoflurane gas (0.5–2.5*%*) with oxygen via a portable field vaporizer, administered initially through a cone shaped mask and afterwards with an endotracheal tube [30]. Once under anesthesia, instruments were attached and physiological samples taken for analysis of oxygen stores [29].

For large-scale tracking we instrumented 10 animals with SPOT5 satellite platform terminal transmitters (PTT) during the warm season (2005), and a total of 8 animals during the cold season (2007), 4 of them with SPOT5 (Wildlife Computers, Richmond, WA, USA) and 4 with Kiwisat 101 PTTs (Sirtrack, Havelock North, New Zealand). To obtain diving behavior data we instrumented sea lions with time-depth recorders (TDR) that sampled every 2 sec, 2 Mk8 and 8 Mk9 models in 2005 and 8 Mk9 in 2007 (Wildlife Computers, Richmond, WA, USA). Sea surface temperature data was also measured and obtained from the TDRs. To locate the animals for instrument recovery when on land we instrumented them with radio transmitters (VHF) (Sirtrack, Havelock North New Zealand).

We mounted instruments on mesh netting and glued them to the dorsal pelage of the lower back and between the shoulders of the animals using 5 minute quick set Loctite epoxy. The total weight of the instruments attached was approximately 230 g (∼0.23*%* of the animal's mass). We weighed animals in a sling using a tripod and a 250 kg (+/− 0.1 kg precision) capacity digital scale and took standard length measurements by using a standard measuring tape. We obtained data from all PTTs and recovered 7 of the 10 TDRs after 15 to 27 days during the warm season (2005), and 5 out of the 8 TDRs after 22 to 89 days during the cold season (2007). Instruments were removed by either physically restraining the animals without anesthesia or had been found on the rookery after molting off. Epoxy mounts fall off within a few months during the animals' annual molt.

#### Galapagos sea lions (GSL)

The same methodology was applied to study the diving behavior of GSL. Research was carried out during a warm season- March 2005 and a cold one- August-September 2006 at Caamaño Islet (0.759°S, 90.278°W) in the Galapagos Islands. During the warm season we captured 11 GSL, which were suckling small pups, 4 to 5 months after peak pupping season. During the cold season we captured 12 GSL, most of them with bigger and possibly older pups (10 to 11 months after peak pupping season) than the ones from the previous season. Details are presented in Villegas-Amtmann and Costa [29].

### Tracking analyses

Habitat utilization and foraging range were determined from ARGOS location data filtered and interpolated using software written in Matlab 7.4.0 (MathWorks Inc, USA) (IKNOS toolbox). The algorithm uses several criteria to remove unlikely locations: (1) realistic travel speeds of a subject between two fixes (≤10 km h^−1^), (2) change in azimuth between successive fixes, (3) Argos location class and (4) time lapse between two consecutive fixes. We plotted filtered locations using Matlab (The MathWorks Inc, USA). Filtered locations were interpolated every 60 min using a Bezier curve to further perform a Gaussian Kernel analysis with a 5 km grid size [3].

### Diving behavior analyses

We analyzed dive data in Matlab 7.4.0 (The MathWorks Inc, USA) using a custom written dive analysis program (Tremblay, *unpublished*) that allows for a zero offset correction at the surface and the identification of dives based on a minimum depth and duration. Diving data were analyzed following the same methods as in Villegas-Amtmann *et al*. [22]. All Mk8 and Mk9 recorders in both seasons had a 0.5 m depth resolution; except one recorder during the cold season had a 1 m depth resolution and all recorders sampled every 2 sec. The minimum depth considered for a dive was 3 m and the minimum duration was 12 sec.

We discarded ‘porpoising’ or shallow dives (restricted to the upper 5 m) typically exhibited by sea lions when travelling [22] to limit our analysis to foraging (i.e. feeding or search) dives. Data were tested for normality using Kolmogorov-Smirnov one sample tests for homogeneity of variance and log transformed as needed. We compared means using t-tests when the data were normally distributed otherwise we used a Kruskal-Wallis test (K–W). We also compared their mass (kg) and body condition index by dividing mass/standard length [31] by using t-tests.

To explore CSL individual diving behavior variability within each season we performed hierarchical cluster analyses (HCA) using Euclidean distance and average linkage method as described in Villegas-Amtmann et al. (2008). Variables used were: dive depth (m), dive duration (sec), bottom time (sec), descent and ascent rate (m/s), dive rate (dives/hr), post dive interval (PDI) (sec), intra depth zone (IDZ) (provides an index of the tendency to repeatedly dive to a given depth, considering 5 m was the minimum detectable depth for a dive, we applied a user defined zone of ±10 m of the maximum depth of the previous dive, i.e. 5 m above and below the previous depth to calculate IDZ, evidence of benthic diving) [32], number of “wiggles” at the bottom of a dive (number of ascent and descent movements at the bottom of the dive, which can imply foraging behavior) [24], max. dive depth (m), max. dive duration (sec), max. distance traveled from the rookery (km), % time spent on land, % time spent at sea and mass (kg). Hierarchical clustering is ideal for small data sets as in this study [33].

To compare diving behavior between species during each season, we reduced the number of variables with a Principal Component Analysis (PCA) using variables from both species and seasons, using latent root criterion, a correlation matrix of extraction, a minimum Eigen-value of 1.0 and a varimax rotation. This analysis is suitable for these data because the diving variables are strongly correlated [33]. Standardized PCA factor scores were then input as dependent variables in a General Linear Model (GLM), independent variables used were species, season and the interaction term: species-season. All means are presented with a±1 standard deviation (SD).

### Isotope analyses

To further analyze and compare foraging behavior and differences in diet [34] between species and seasons we measured carbon (^13^C/^12^C) and nitrogen (^15^N/^14^N) isotope ratios by collecting blood samples from the caudo-gluteal vein into serum collection tubes. Stable isotopes measured from serum reflect diet incorporated over a time period of days before sampling [35], [36]. Serum was separated by centrifugation and stored at −20°C. Serum samples were freeze-dried; and homogenized after lipid extraction. Samples were analyzed using a Carlo Erba 1108 Elemental Analyzer coupled to a Thermo Finnigan Delta Plus XP isotope ratio mass spectrometer (Stable Isotope Laboratory, University of California Santa Cruz). Stable isotopes values are given in delta notation (δ) as parts per thousand (‰). Data were tested for normality using Kolmogorov-Smirnov one sample tests for homogeneity of variance and log transformed as needed. We compared foraging behavior of each species between seasons by running an ANOVA.

Significance was tested at the 95% confidence. All statistical analyses were performed in SYSTAT 10.2 and/or 11.

## Results

### California sea lion seasonal diving behavior

CSL exhibited changes in their dive behavior between seasons, during the warm season CSL dived significantly deeper (t-test, *t* = −2.24, *df = *9.7, *P* = 0.05), longer (t-test, *t* = −4.31, *df* = 9.9, *P*<0.01), spent significantly longer time at the bottom of a dive (t-test, *t* = −3.95, *df* = 8.9, *P*<0.01), presented significantly greater maximum dive durations (t-test, *t* = −2.86, *df* = 7.7, *P* = 0.02), percentage of intra-depth zone (IDZ) dives (t-test, *t* = −4.39, *df* = 10.0, *P* = 0.001), and significantly lower percentage of time spent at sea (t-test, *t* = 4.80, *df* = 8.2, *P* = 0.001) compared to the cold season (Table 1). Maximum distance traveled, maximum dive depth, descent and ascent rate, dive rate and post-dive interval (PDI) were not significantly different between seasons (Table 1).

**Table 1:** California sea lion dive parameters.

Female ID	Mean dive depth (m)	Max. dive depth (m)	Mean dive duration (min)	Max. dive duration (min)	Mean bottom time (min)	Mean # bottom wiggles	Mean descent rate (m/s)	Mean ascent rate (m/s)	Mean dive rate (dives/hr)	PDI (min)	% IDZ	Max. dist. traveled (km)	% time on land	% time at sea	% time diving at sea
**Warm Season**															
GF2	46.7±32.7	240.5	3.4±1.6	9	1.5±1.0	4.8±3.7	0.9±0.3	0.7±0.3	9.6±5.1	9.6±141.0	43.8	48.3	59.1	40.9	52.66
GF3	70.1±49.2	284	3.1±1.2	8.1	1.2±0.8	4.4±3.7	1.0±0.6	1.1±0.6	10.4±6.0	9.6±141.2	58.6	87.9	60.1	39.9	50.33
GF6	113.5±76.0	295	4.2±1.7	8.8	1.5±0.8	5.3±3.7	1.3±0.5	1.1±0.6	6.7±3.7	13.4±183.1	62.6	49.2	54.7	45.3	40.96
GF8	78.8±59.7	324	3.0±1.4	7.8	1.0±0.8	3.6±3.1	1.2±0.6	1.1±0.7	8.2±4.8	8.3±88.8	49.6	51.6	56.3	43.7	44.46
GF10	106.0±70.8	288	4.5±2.1	9	2.0±1.3	4±4.4	1.2±0.5	1.1±0.5	5.2±2.6	15.6±141.4	55.2	96.6	43.8	56.2	36.2
GF4	117.8±49.4	194.5	4.6±1.4	8.1	2.2±1.0	6.4±3.7	1.5±0.5	1.5±0.5	7.7±3.5	6.0±68.6	80.7	84.2	37.9	62.1	55.82
GF5	38.9±22.6	140	2.1±0.9	5.8	0.8±0.6	4.7±3.9	1±0.4	1.0±0.4	12.3±8.9	4.4±48.1	54.9	91	57.6	42.4	42.99
**Cold Season**															
GFC1	35.7±48.1	243	1.4±1.2	7.2	0.6±0.5	3.0±0.05	1.0±0.5	1.1±0.6	7.7±8.4	1.7±25.9	32.1	162.5	37.1	62.9	47.95
GFC2	43.9±64.0	242	1.4±1.6	6.6	0.4±0.6	3.3±3.7	1.0±0.6	1.1±0.6	9.6±9.9	5.7±44.4	26	59	35.6	64.4	23.16
GFC9	50.3±64.3	217	1.6±1.5	6.3	0.5±0.6	3.4±3.7	1.1±0.6	1.1±0.5	8.4±9.8	7.5±55.2	31.1	81.35	40.6	59.4	21.43
GFC10	80.0±66.5	220.5	2.7±1.5	7.1	1.0±0.7	5.8±4.7	1.3±0.7	1.3±0.6	8.0±6.9	8.3±55.3	46.3	97.5	33.8	66.2	33.73
GFC11	34.7±54.0	244.5	1.3±1.6	6.8	0.5±0.7	4.2±5.1	0.9±0.5	1.1±0.5	14.3±19.1	2.8±21.2	30.7	105.7	32.2	67.8	30.47

PDI = post-dive interval, IDZ = intra-depth zone dives.

Mean (±SD) and maximum dive parameters of individual female California sea lions (*Zalophus californianus*) from Granito Island, Mexico recorded for 15–27 days during a warm season (Jul–Aug 2005) (mean no. dives 2068±655) and for 22–89 days during a cold season (Feb–Mar 2007) (mean no. dives 8322±9511). Female IDs organized by groups produced by the cluster analysis.

**Figure 1:**
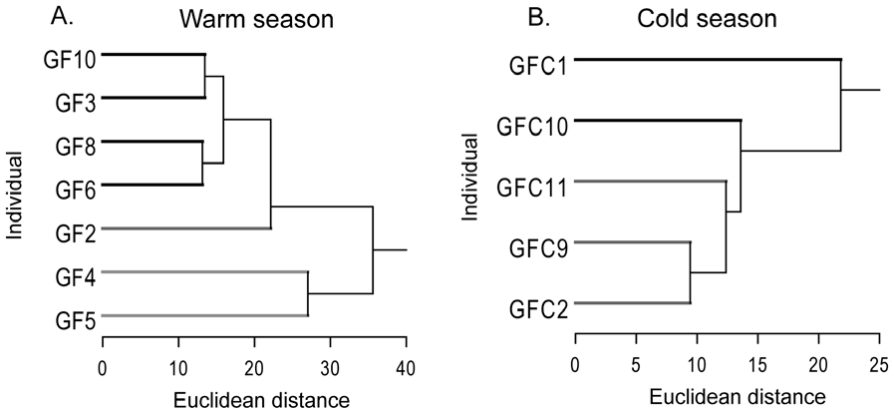
California sea lions diving behavior cluster analyses. California sea lion females (*Zalophus californianus*) from Granito Island, Mexico, cluster analyses for dive parameters during a warm season (Jul–Aug. 2005) and a cold season (Feb–Mar. 2007).

CSL dive behavior exhibited greater variability during the warm season compared to the cold one as observed in the three groups or foraging strategies identified in the cluster tree for the warm season compared to no groups observed during the cold season (Fig. 1). The maximum Euclidean distance for a group to be considered was 27 based on the cluster tree produced by the HCA (Fig. 1). Due to our small sample size we ran a cluster analysis with data combined from both seasons. The cluster tree obtained produced 3 groups with all individuals from the cold season in one and individuals from the warm season in the 3 different groups, confirming our previous findings.

#### Gulf of California environmental conditions

SST encountered by sea lions was significantly greater during the warm (mean SST for each individual sea lion, 28.8±1.1°C) than the cold season (17.5±0.9°C, K–W test, *x^2^* = 6.82, *df* = 1, *P* = 0.01).

### California and Galapagos sea lion seasonal diving behavior comparison

#### Principal Component Analysis (PCA)

Four factors or principal components (PC) explained 86% of the variance. The variables driving the 4 PC were: PC1- dive rate, dive depth, max. dive depth and PDI; PC2- Bottom time, % IDZ dives, bottom wiggles (number of ascent and descent movements at the bottom of the dive, which can imply foraging behavior) [24] and dive duration; PC3- percent time spent on land and at sea and PC4- mass and body condition index (Table 2).

**Table 2:** Galapagos and California sea lions dive parameters PCA loading matrix.

Dive Parameter	PC1 (29.06%)	PC2 (26.73%)	PC3 (15.67%)	PC4 (14.53%)
Dive depth	**0.858**	0.36	0.142	−0.06
Dive duration	0.645	**0.741**	0.083	0.005
Bottom time	0.283	**0.909**	0.155	0.057
Max. dive depth	0.866	−0.271	0	−0.053
Max. dive duration	0.689	0.441	0.115	−0.128
Max. distance traveled	0.231	−0.669	0.297	0.24
Dive rate	**−0.894**	−0.11	−0.071	0.11
PDI (Post dive interval)	**0.829**	0.105	−0.201	−0.137
%IDZ (Intra-depth zone dives)	0.09	**0.886**	0.043	0.148
Bottom wiggles	0.13	**0.829**	0.305	0.008
% time on land	−0.008	−0.11	**−0.965**	0.167
% time at sea	0.001	0.11	**0.968**	−0.104
Mass	−0.144	0.037	−0.139	**0.961**
Body condition index	−0.164	0.021	−0.121	**0.967**

PCA rotated loading matrix for Galapagos and California sea lion dive parameters from Granito Island, Mexico and Caamaño Islet, Galapagos for a warm (GSL, Mar.2005 and CSL, Jul–Aug.2005) and cold season (GSL, Feb–Mar.2006 and CSL, Feb–Mar.2007). In bold are loadings from the diving variables that contributed the most for Principal components (PC) 1–4.

#### General Linear Model (GLM)

**Figure 2:**
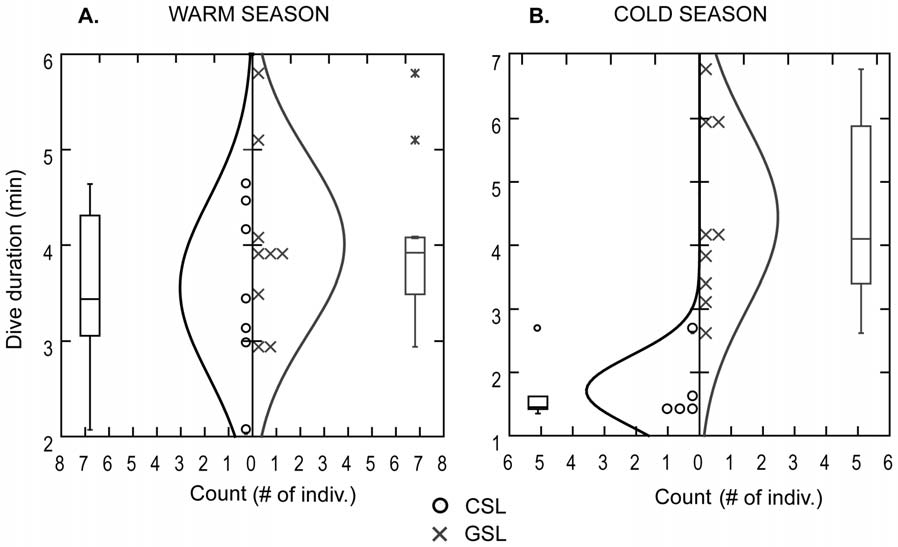
California (CSL) and Galapagos (GSL) sea lions dive durations. Dive duration frequency distribution plots for California (CSL, *Z. californianus*) and Galapagos (GSL, *Z. wollebaeki*) sea lion females during a warm (CSL, Jul–Aug. 2005; GSL, Mar. 2005) and a cold (CSL, Feb–Mar. 2007; GSL, Aug–Sep. 2006) season.

GLM results showed that dive depth, dive rate, maximum dive depth & PDI (PC1) were not significantly different between species, seasons or species-season (interaction term). This result remained even after eliminating the interaction term from the model. Dive duration, bottom time, bottom wiggles & IDZ (PC2), were significantly greater for GSL than CSL (*df* = 1, *F-ratio* = 34.65, *P*<0.001) and significantly greater during the warm season compared to the cold one in CSL (interaction term: species-season) (*df* = 1, *F-ratio* = 10.69, *P*<0.001). GSL exhibited no change in these dive parameters between seasons (Table 3 & Fig. 2). Percent time on land and at sea was significantly different between seasons (*df = *1, *F-ratio* = 15.17, *P*<0.01) and between seasons and species (interaction term) (PC3)) (*df* = 1, *F-ratio* = 12.34, *P*<0.01). During the warm season CSL spent more time on land (52.8±8.5%), conversely GSL spent more time at sea (59.2±5.1%) (Table 3). Mass and body condition index (PC4) were not significantly different in the GLM. Therefore, we excluded the interaction term (seasons-species) from the model and found that CSL mass and body condition index were significantly greater than GSL (*df* = 1, *F-ratio* = 4.32, *P* = 0.05).

**Table 3:** Galapagos and California sea lions dive parameters.

	WARM SEASON				COLD SEASON			
	GSL (N = 9)		CSL (N = 7)		GSL (N = 9)		CSL (N = 5)	
	Mean ±SD	Range	Mean ±SD	Range	Mean ±SD	Range	Mean ±SD	Range
**Dive depth (m)**	91.8±35.2	45–149	81.7±31.9	46.7–117.8	92.7±46.8	30.4–192.6	48.9±18.5	34.7–80.0
**Dive duration (min)**	4.0±0.9	2.9–5.8	3.5±0.9	2.1–4.6	4.4±1.4	2.6–6.8	1.7±0.6	1.3–2.7
**Bottom time (min)**	1.9±0.6	1.1–2.9	1.5±0.5	0.8–2.2	2.3±0.8	1.1–3.6	0.6±0.2	0.4–1.0
**# Bottom wiggles**	8.4±4.8	2.1–17.5	4.7±0.9	3.6–6.4	12.8±4.9	5.7–20.3	3.9±1.1	3.0–5.8
**Descent rate (m/s)**	1.2±0.1	1.1–1.5	1.2±0.2	0.9–1.5	1.4±0.2	0.8–1.6	1.0±0.1	0.9–1.3
**Ascent rate (m/s)**	1.2±0.1	1.0–1.4	1.1±0.2	0.7–1.5	1.2±0.4	0.1–1.6	1.1±0.1	1.1–1.3
**Dive rate (dives/hr)**	8.4±2.7	5.2–13.5	8.0±2.4	5.2–12.3	8.4±3.1	4.3–13.6	9.6±2.7	7.7–14.3
**% IDZ dives**	60.3±18.8	31.3–88.9	57.9±11.8	43.8–80.7	69.4±19.7	29.0–89.0	33.3±7.7	26.0–46.3
**PDI (min)**	6.3±2.5	2.7–10.9	9.0±4.1	4.4–15.6	7.4±3.4	3.3–13.9	5.2±2.9	1.7–8.3
**Max. dist traveled (km)**	41.8±20.3	14.3–76.2	72.7±21.8	48.3–96.6	49.0±18.7	8.5–75.4	101.2±38.6	59–162.5
**Max. dive depth (m)**	233.4±110.1	84.5–371	252.3±65	140–324	241.4±97.3	71–387	233.4±13.5	217–244.5
**Max. dive duration (min)**	8.5±1.3	6.4–9.8	8.1±1.1	5.8–9	9.4±1.2	7.5–11.1	6.8±0.4	6.3–7.2
**% time on land**	40.8±5.1	31.7–46.6	52.8±8.5	37.9–60.1	40.6±6.7	30.9–49.7	35.9±3.2	32.2–40.6
**% time at sea**	59.2±5.1	53.4–68.3	47.2±8.5	39.9–62.1	59.3±6.7	50.3–69.1	64.1±3.2	59.4 - 67.8
**% time diving at sea**	52.9±5.1	28–70.4	46.2±7.0	36.2–55.8	47.5±19.5	41.6–67.6	31.3±10.6	21.4–47.9

Dive parameters of Galapagos (GSL) and California (CSL) sea lions from Granito Island, Mexico and Caamaño Islet, Galapagos for a warm (GSL, Mar.2005 and CSL, Jul–Aug.2005) and cold season (GSL, Feb–Mar.2006 and CSL, Feb–Mar.2007).

#### Gulf of California and Galapagos environmental conditions

During the warm season, SST (mean SST for each sea lion) encountered by CSL (29.2±0.6°C, *n* = 5, t-test, *t* = 8.13, *df* = 8, *P*<0.001) was significantly higher than that encountered by GSL (26.7±0.4°C, *n* = 5). During the cold season, SST encountered by CSL (17.5±0.9°C, *n* = 5, t-test, *t* = −8.68, *df* = 12, *P<*0.001) was significantly lower than that encountered by GSL (23.3±1.3°C, *n* = 9). During the cold season GSL encountered mild El Niño conditions (http://coastwatch.pfeg.noaa.gov/).

### California and Galapagos sea lions foraging areas

**Figure 3:**
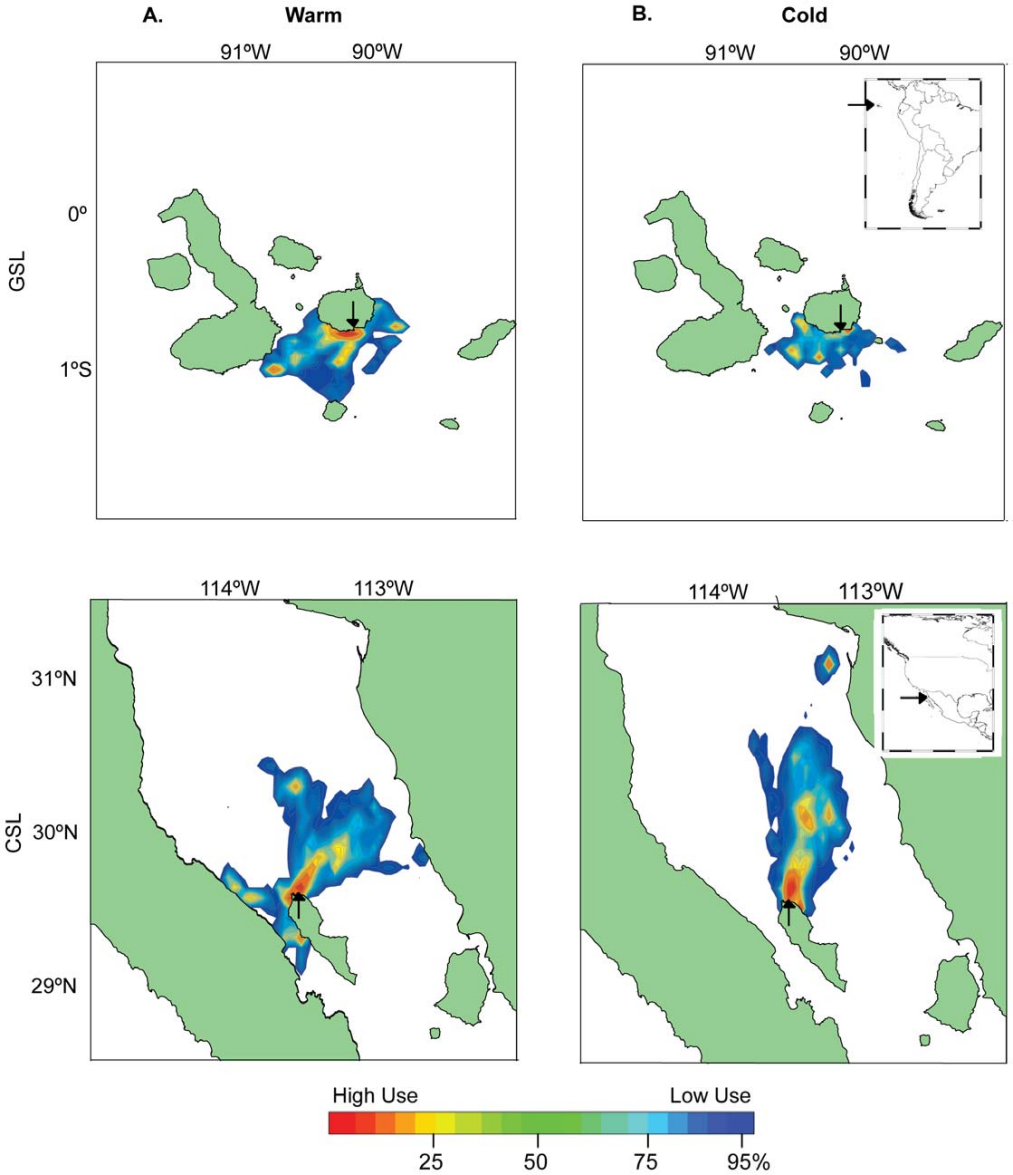
Galapagos and California sea lions' foraging areas. Kernel analyses of Galapagos (GSL, *Z. wollebaeki*) and California (CSL, *Z. californianus*) sea lion females' foraging areas during a warm (CSL, Jul–Aug. 2005, n = 10; GSL, Mar. 2005, n = 9) and a cold (CSL, Feb–Mar. 2007, n = 8; GSL, Aug–Sep. 2006, n = 10) season. Black arrow shows colony location.

The kernel analysis revealed that CSL utilized a greater diversity of foraging areas during the warm season (*n* = 10 females) compared to the cold one (*n* = 8 females). GSL exploited the same foraging areas during both seasons (*n* = 9 & 10 females respectively) (Fig. 3).

### Isotope analyses

**Figure 4:**
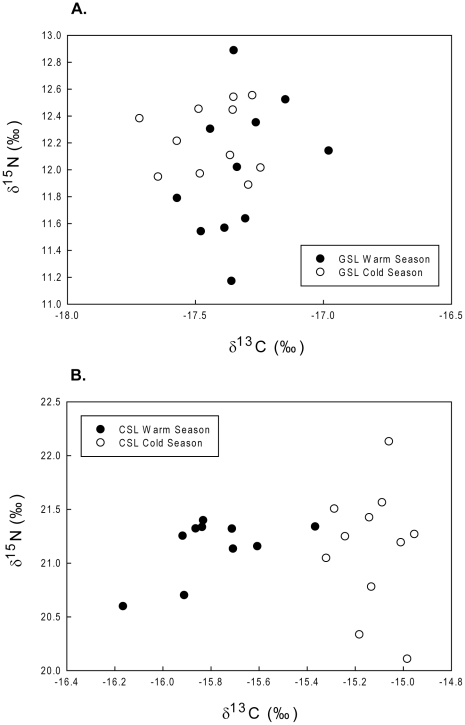
Galapagos and California sea lions δ^13^C and δ^ 15^N values. δ^13^C and δ^ 15^N values of Galapagos (GSL, *Z. wollebaeki*) and California (CSL, *Z. californianus*) sea lion females for a warm (CSL, Jul–Aug. 2005, n = 10; GSL, Mar. 2005, n = 11) and a cold (CSL, Feb–Mar. 2007, n = 11; GSL, Aug–Sep. 2006, n = 11) season.

ANOVA tests on sea lionś δ^13^C and δ^15^N isotope ratios showed that CSL δ^13^C values were significantly different (*df* = 1, *F-ratio* = 77.43, *P*<0.001) between seasons, while δ^15^N values were not (*df = *1, *F-ratio* = 0.001, *P* = 0.97). GSL δ^13^C and δ^15^N values were not significantly different between seasons (*df* = 1, *F-ratio* = 2.61, *P = *0.12 and *df* = 1, *F-ratio* = 1.97, *P* = 0.17 respectively) (Fig. 4).

## Discussion

### CSL foraging behavior

CSL exhibited changes in their dive behavior between seasons. As hypothesized, CSL showed greater effort during the warm and less productive season [37] as reflected in significantly greater dive duration, bottom time and bottom wiggles compared to the cold season (Table 1 & Fig.2). During the warm season, CSL diving parameters, foraging areas and stable isotopes results (i.e. diet) showed greater individual variability and foraging specialization [37] possibly caused by increased intra-specific competition in less productive waters (Fig.1 & 3). This intra-specific competition during the warm and less productive season, appears to relax during the cold season when resource availability increases [37], as observed in their reduced foraging effort and variability during this season.

### Species comparison

CSL and GSL respond differently to environmental variability. CSL exhibit greater variability in their diving behavior during the warm season (Table 1, Fig.2 & 3) when resources are limited compared to the cold and more productive season [37]. This diving behavior variability is confirmed by the results obtained from the stable isotope analysis. The high predictability in δ^15^N's fractionation between trophic levels (∼ +3‰ per trophic step in marine ecosystems) makes it a good indicator of the trophic position of consumers, while δ^13^C, despite being less useful to indicate trophic level, is largely recognized to be a very good indicator of the food sources (i.e. prey items) and habitats utilized by the consumer [34], [38]. Accordingly, we did not find seasonal differences in δ^15^N of CSL, showing that they feed on the same trophic level between seasons but with greater trophic diversity during the cold season. These results are supported by Garcia-Rodriguez and Aurioles (2004) findings [39]. However, their δ^13^C signatures show variability in their feeding sources (prey items), which is supported by their spatial distribution and diving behavior during both seasons (i.e. pelagic vs. benthic or pelagic vs. coastal during the warm and cold season respectively) [40] (Fig. 4). Correspondingly the diet of CSL at Granito Island has been shown to differ temporally; feeding primarily on Pacific cutlassfish (*Trichiurus lepturus*) in September and on sardine (*Sardinops caeruleus*), sanddab (Citharichthys sp.) and mictophids in January [39].

In contrast, GSL do not exhibit a change in dive behavior between seasons [29] presenting great individual variability reflected in 3 foraging strategies observed during both seasons. This persistence in foraging behavior between seasons is also revealed in their δ^15^N and δ^13^C isotopic signatures as no change was observed between seasons. GSL are likely feeding on the same trophic level between seasons (Fig. 4). GSL diet changes temporally as expected from changes in primary productivity, during the warm season they feed primarily on snake eels (*Ophichthus sp*.), herring (*Opisthonema sp*.) and fish from the family Sciaenidae, and during the cold season they feed on myctophids, sardines (*Sardinops sp*.) and fish from the family Ophididae [41]. Although primary productivity changes seasonally in the Galapagos Archipelago, GSL individual specialization suggests the persistence of intra-specific competition throughout seasons as reflected in the diversity of foraging areas utilized (Fig. 3). This competition indicates that resources might be limited year-round in this area of the Galapagos, which has been described as a low productivity system [42], [43]. These results are consistent with foraging observation on sea otters that suggests that intra-specific competition for limited resources is an ecological prerequisite for foraging specializations [44].

### Latitudinal range implications

Sea lions living in equatorial regions face a more unpredictable oceanic system with lower productivity and lower range of variation than sea lions living at higher latitudes. Hence GSL do not express plasticity in diving behavior between seasons suggesting that when living in a system with low productivity year round individuals are always competing. Additionally, GSL encounter stronger El Niño events that lead to even lower productivity. In contrast, CSL encounter greater but predictable environmental change (SST and chl-a) in the form of seasonal variation. Therefore, while CSL can adjust their behavior to predictable seasonal changes in productivity, the overall lower productivity and environmental unpredictability faced by GSL makes adjustments in their behavior challenging, favoring individual prey specialization and probably making them more vulnerable to climate change [45].

CSL spent significantly more time on land than at sea with greater dive effort during the warm season when their pups are young, when rearing demands are greatest. In contrast, GSL exhibited no change in the percent of time they spent on land and at sea. Body condition was significantly lower in GSL than CSL especially during the warm season when mean SST encountered by CSL was greater, ruling out a possible cause of greater insulation due to colder temperatures. These data suggest that compared to CSL, GSL are limited in their ability to invest resources in pup rearing because although productivity is seasonally variable, it is absolutely lower in the Galapagos. This constraint generates higher nutritional stress in GSL pups reflected in a pup survival difference between the two species. CSL pup survival rates range from 0.556 to 0.998 between different years [46] while GSL pup survival rate is estimated to be between 0.55 and 0.91, with females successfully rearing pups only every other year and on average for the population at Caamaño Islet only one pup every three years (Müller, *personal communication*).

As seen in other terrestrial and marine species [10]–[12], GSL living in equatorial, less variable and less productive environments [37], [47], [48], exhibit greater foraging effort, lower foraging success (lower body condition index), and their pups face higher nutritional stress compared to CSL living in temperate regions. These differences likely influence population growth and are likely to be contributing factors in the different population status of the 2 species: GSL population being smaller, endangered and possibly with a greater risk of extinction [12] compared to CSL population that is larger, increasing and more widely distributed.

### Conservation implications

While the total population of CSL is increasing [13], the population at the Gulf of California is decreasing [49] and as a consequence problems with fisheries interactions become more critical. California sea lions in the upper region of the Gulf of California obtain the main portion of their diet from a relatively small number of species and the decrease in abundance of any of these food resources can seriously affect their population [39].

The GSL population is endangered and declining [16] and is greatly affected by El Niño events [50]. There has been a 50% population decline over the last 30 years [51] and one of its main conservation concerns is also the interaction with fisheries [52]. Furthermore, recent studies have shown that *Z. wollebaeki* is an important vector for the transport of marine nutrients to the terrestrial ecosystem [53]. Therefore its conservation is vital.

As these issues become more evident, knowledge of these sea lions critical habitat is crucial. Data presented here provides information of their habitat utilization, diving behavior, and foraging areas, and could potentially facilitate the creation of protected areas with regulated fishing activities for their future protection and conservation.
